# Preserved Ejection, Lost Rhythm: A Narrative Review of the Pathophysiology and Management of Heart Failure with Preserved Ejection Fraction and Concomitant Atrial Fibrillation

**DOI:** 10.3390/jcm15030969

**Published:** 2026-01-25

**Authors:** Andrea Ballatore, Alan Poggio, Andrew P. Sullivan, Andrea Saglietto, Gaetano Maria De Ferrari, Matteo Anselmino

**Affiliations:** 1 Division of Cardiology, Cardiovascular and Thoracic Department, “Città della Salute e della Scienza” Hospital, 10126 Turin, Italyalan.poggio@edu.unito.it (A.P.);; 2 Department of Medical Sciences, University of Turin, 10124 Turin, Italy; 3 UBC Division of Cardiology, Gordon & and Leslie Diamond Health Care Centre, 2775 Laurel St., 9th Floor, Vancouver, BC V5Z 1M9, Canada

**Keywords:** atrial fibrillation, HFpEF, catheter ablation

## Abstract

Atrial fibrillation (AF) and heart failure with preserved ejection fraction (HFpEF) coexist in 40–60% of cases and mutually reinforce each other through adverse electrical, cellular, and functional remodelling. There is considerable overlap in signs and symptoms, and diagnosis may be challenging due to nonspecific clinical presentations and chronic course. AF is clearly linked with worsening morbidity and mortality in HFpEF with higher rates of HF hospitalizations, HF progression, stroke, systemic embolism, and all-cause death. Optimal management of HFpEF-AF patients requires aggressive treatment of comorbidities and risk factor modification. Sodium-glucose cotransporter 2 (SGLT2) inhibitors have demonstrated consistent benefit with respect to HF hospitalizations, symptoms and exercise haemodynamics, and potential to reduce AF burden. Gastric inhibitory polypeptide (GIP)/glucagon-like peptide-1 (GLP-1) agonists, mineralocorticoid receptor antagonists (MRAs), angiotensin receptor-neprilysin inhibitors (ARNIs), and statins may provide benefit in selected phenotypes, though evidence remains heterogeneous. A rhythm control strategy in the early clinical course of HFpEF might be a reasonable strategy to improve symptoms and delay both AF and HFpEF disease progression. Catheter ablation appears to improve exercise haemodynamics and quality of life, and observational data suggest it may reduce mortality and HF hospitalization, though current evidence is inconsistent and not yet definitive. Emerging device-based and molecular therapies could represent promising avenues for future research. Overall, early detection of AF, comprehensive risk-factor modification, and tailored rhythm-control strategies are central to improving outcomes in the HFpEF-AF overlap syndrome.

## 1. Introduction

Atrial fibrillation (AF) and heart failure with preserved ejection fraction (HFpEF) are highly prevalent in the general population and frequently coexist. Together, these conditions reciprocally give rise to a vicious cycle altering the electrophysiologic properties of atrial and ventricular myocardial structure and function, while also adversely affecting patients’ quality of life and overall survival [1,2,3]. HFpEF goes undiagnosed in roughly one-third of patients presenting with exertional dyspnoea and fatigue [4], and is projected to become the most prevalent HF phenotype, affecting 1 in 10 adults [5]. This narrative review provides clinicians with an up-to-date overview of the clinical features, challenges, and opportunities in this increasingly important clinical scenario.

## 2. Epidemiology

AF is the most common sustained tachyarrhythmia, with about 52.6 million individuals affected in 2021 [6]. Heart failure (HF) has a global prevalence exceeding 64 million individuals, and HFpEF constitutes approximately half of these cases [2]. The lifetime risk of HF is greater than 10% for both men and women by age 45 years [7], and up to 4.9% of individuals over 60 years of age are afflicted by this condition [8]. While all-cause mortality and total hospitalization rates are similar between HFpEF and HF with reduced ejection fraction (HFrEF), HFpEF patients have a lower risk of cardiovascular mortality and hospitalizations for heart failure [9]. Alarmingly, both AF and HFpEF are projected to continue rising, with AF predicted to reach an estimated 12.1 million affected subjects by 2030 [10]. Similarly, HFpEF-related hospital admissions more than doubled in the United States from 2008 to 2018, with over 495,000 subjects requiring hospitalization [11].

## 3. Definitions

In 2021, a universal definition of HF was established, defining HFpEF as a clinical syndrome with a left ventricular ejection fraction (LVEF) ≥ 50%, together with elevated natriuretic peptide (NP) levels and/or other objective evidence of lung or systemic congestion [12] in symptomatic patients.

Nevertheless, this definition carries two main limitations. Firstly, several cardiac and non-cardiac conditions may give rise to a clinical syndrome similar to HFpEF, despite important differences in diagnosis, pathophysiology, and management (e.g., amyloidosis, sarcoidosis and other infiltrative diseases, hypertrophic cardiomyopathy, valvular or pericardial diseases, and pulmonary arterial hypertension [5]). Secondly, due to clinical compensation, signs and symptoms of congestion might frequently be absent in apparently healthy ambulatory patients complaining of exertional dyspnoea or fatigue. Moreover, plasmatic NP levels can be within normal ranges in about 30% of HFpEF patients, especially those with obesity-related HFpEF, leading to a systemic underestimation of congestion [13].

Because of the heterogeneity of HFpEF as a clinical syndrome, a classification based on four main phenogroups has been proposed, with each group characterized by shared functional and structural anomalies, but with differences in their pathophysiology, impact on haemodynamics, and management (Table 1) [14]. Importantly, these subgroups should not be viewed as discrete entities. Two-thirds of individuals with HFpEF meet criteria for more than one phenogroup, and this overlap is associated with a 94% higher risk of death and a 74% higher risk of HF-related hospitalization compared to those assigned to a single phenogroup [14].

## 4. Diagnostic Criteria

**Figure 1:**
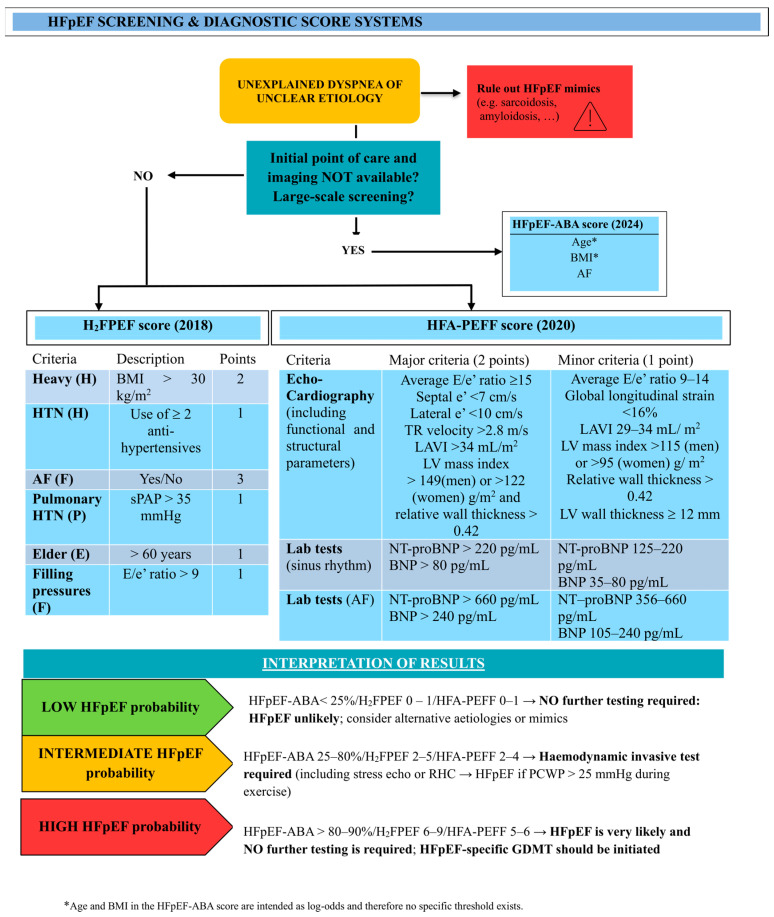
Screening and diagnostic HFpEF score systems: HFpEF-ABA Score (2024) [19], H_2_FPEF score (2018) [20], and HFA-PEFF score (2020) [21]. This figure was inspired by Borlaug et al. (Figure 7 in [5]). Abbreviations: as in Table 1. New abbreviations: HTN = Hypertension; sPAP = Pulmonary artery systolic pressure; PCWP = Pulmonary Capillary Wedge.

HFpEF remains underdiagnosed in clinical practice due to the low specificity of its symptoms and its progressive course. As a result, different scores have been created and validated to guide physicians and orient the choice for more invasive testing (Figure 1). The hallmark for the diagnosis of HFpEF is elevated left atrial (LA) pressures (>15 mmHg at rest), together with typical symptoms of HF, including dyspnoea and fatigue [4]. Indeed, the diagnostic gold standard remains invasive assessment of pulmonary capillary wedge pressure (PCWP) during right heart catheterization (RHC). Nevertheless, since up to one third of patients present with normal LA pressure at rest, invasive exercise RHC should be performed in case of diagnostic uncertainty, albeit being available in a limited number of centres. Saline infusion and passive leg raise may be used to unmask a reduced tolerance to stress and elevated LA pressure, but they are not equivalent to invasive exercise testing. Moreover, because both AF and HFpEF often share common symptoms (i.e., exertional dyspnoea and fatigue), the diagnosis of HFpEF is harder to make solely based on clinical features. Additionally, the absence of atrial contraction and beat-to-beat variability due to AF can impair the interpretation of diastolic function on echocardiography [15]. Interpretation of NP levels in patients with AF is challenging, since both AF and HFpEF are independently associated with increased NP concentrations. Higher diagnostic thresholds for BNP/NT-proBNP are therefore recommended when AF is present (see Figure 1) [16] and have been adopted in the HFA-PEFF score. This relationship is further complicated in patients with obesity, as this condition is associated with restrictive physiology and lower NP levels. Accordingly, the HFA-PEFF score, relying on higher NP thresholds and LA volumes indexed to body surface area, shows reduced sensitivity in the subset of patients suffering from both AF and obesity [17].

Similarly, assessment of diastolic function in AF patients has been traditionally cumbersome due to the irregular rhythm and the inability to evaluate the E/A ratio. A recent consensus document recommends first evaluating E velocity and deceleration time, septal E/e’ ratio, and tricuspid regurgitation velocity; in the presence of intermediate findings, additional parameters should be assessed, including the BMI of the patient and LA strain. The latter, in particular, is a promising technique for identifying subclinical alterations in LA structure and function [18]. Moreover, up to one-third of patients may exhibit normal hemodynamic values at rest, with abnormal parameters manifesting only during exercise; in these patients, typically presenting with exertional dyspnea and a high clinical suspicion of HFpEF after exclusion of other potential causes, invasive exercise testing should be considered [5].

Finally, the accuracy of diagnostic scoring systems like H_2_FPEF is reduced in the setting of AF, since the presence of AF may disproportionately elevate the final score, potentially overestimating the pre-test probability of HFpEF [15].

## 5. Pathophysiology of HFpEF

**Figure 2:**
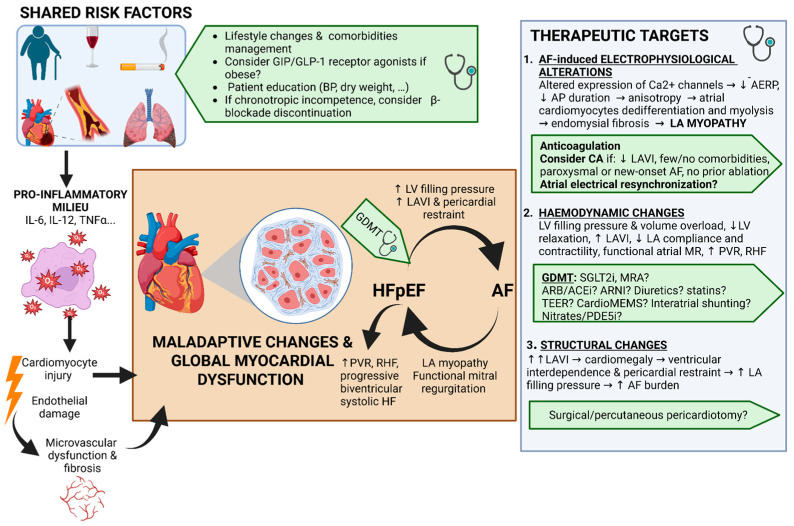
Pathophysiology and therapeutic targets in the negative interplay between HFpEF and AF. Abbreviations as in Table 1 and Figure 1. ↓ = decrease(d) or lower; ↑ = increase(d) or higher. Others: BP = Blood Pressure; ROS = Reactive Oxygen Species; AERP = Atrial Effective Refractory Period; AP = Action Potential, MR = Mitral regurgitation, PVR = Pulmonary Vascular Resistance; RHF = Right Heart Failure; CA = Catheter Ablation; GDMT = Guidelines-Directed Medical Therapy; SGLT2i = Sodium-Glucose Cotransporter 2 Inhibitors; MRA = Mineralocorticoid Receptor Antagonists; ARB = Angiotensin Receptor Blockers; ACEi = Angiotensin Converting Enzyme Inhibitors; ARNI = Angiotensin Receptor Blocker and Neprilysin Inhibitor; PDE5i = Phosphodiesterase-5 inhibitors.

Most risk factors are shared between HFpEF and AF, and each condition can both precipitate and exacerbate the other [3,22]. HFpEF arises when impaired LV compliance leads to elevated filling pressures. Traditionally, this dysfunction was attributed to a sustained increase in afterload (typically from systemic hypertension), driving LV hypertrophy and diastolic dysfunction. However, HFpEF develops not only in the setting of hypertension but also in association with comorbidities such as obesity, diabetes mellitus (DM), obstructive sleep apnoea syndrome (OSAS), smoking, excessive alcohol consumption, chronic renal disease (CKD), iron deficiency, and coronary artery disease (CAD) [22,23]. Over time, these conditions adversely affect both the atrial and ventricular myocardium, fostering an inflammatory milieu characterized by reactive oxygen species, oxidative stress, endothelial dysfunction, and microvascular inflammation [24]. Figure 2 summarizes these mechanisms. It is also believed that the accumulation of epicardial adipose tissue (EAT), especially in the cardiometabolic phenotype of HFpEF, contributes to myocardial inflammation and fibrosis, as well as exerting an excessive extrinsic pericardial restraint on the heart [25,26,27]. Finally, all these processes have haemodynamic consequences and can progress to LA myopathy, and subsequently AF, pulmonary hypertension, and culminate in right ventricular (RV) failure [28,29].

## 6. AF, LA Myopathy and HFpEF Interplay

According to the principle that “AF begets AF” [30], new-onset AF leads to metabolic changes, including increased intracellular calcium, reduced L-type calcium currents, shortened action potential duration, and reduced refractoriness, promoting multiple disorganized atrial reentrant wavelets [31,32,33]. Within weeks of sustained AF, atrial function declines due to myocyte dedifferentiation and hypertrophy, cytological alterations, and myolysis. Irregular atrial activation also increases oxidative stress, damages DNA, alters ion channel expression, and promotes fibrosis, while fibroblasts can impair impulse propagation [34,35,36]. This self-perpetuating process supports the concept that early rhythm control may limit progression and reduce AF burden [37].

In HFrEF patients, AF is frequent and can exacerbate the progression of heart failure. Indeed, the irregular heart rhythm in AF increases the ventricular rate, leading to worsened hemodynamics and worsening left ventricular function. This dynamic creates a vicious cycle, worsening symptoms and prognosis in affected patients. Similarly, the interplay between AF and HFpEF is strong and bidirectional. AF can worsen HFpEF through loss of atrial systole, functional mitral regurgitation, impaired calcium handling, tachycardia, reduced ventricular filling, and tachycardia-mediated cardiomyopathy. Neurohumoral activation further promotes myocardial dysfunction [11,38,39,40]. Conversely, HFpEF promotes AF through chronically elevated filling pressures, atrial stretch, fibrosis, mitral annular dilation, and increased atrial wall stress [5,41,42,43]. Disease progression follows from paroxysmal to permanent AF, pulmonary vascular remodelling, and RV dysfunction.

In addition, atrial myopathy, characterized by a derangement of atrial function and profound alterations in its structure, can precede or be caused by AF onset. The LA myopathy is out of keeping with LV dysfunction and is the primum movens in the disease progression. Atrial myopathy causes an inability to augment the stroke volume in response to increases in preload. This reduced LA compliance may impair dynamic responses and physiological adaptations during hemodynamic changes, as during exercise. This subset of patients is at higher risk of AF development, but the arrhythmia does not appear to be associated with the observed hemodynamic changes [44]. Moreover, specific anatomical subtypes of LA remodelling have been observed in patients with elevated LA pressure at rest and during exercise—the latter demonstrates overall better LA function and lower LA dilation, but shows an eccentric LA remodelling pattern with dilation in the LA roof [45,46].

AF and HFpEF coexist in 40–60% of cases [3,47] and an estimated 40% of patients with HF develop AF [48]. An analysis from the Framingham Heart Study found that, among individuals with newly diagnosed HF, AF was more prevalent in HFpEF patients compared to those with HFrEF (32% versus 23%, respectively; *p* = 0.002). AF was also more likely to be newly diagnosed in HFpEF patients (62% in HFpEF vs. 55% in HFrEF; *p* = 0.02) [3]. Conversely, 50% of patients with a known AF diagnosis developed HFpEF (hazard ratio—HR: 2.34, 95% CI: 1.48–3.70), while only 40% developed HFrEF. Yet another study assessing symptomatic AF patients referred for catheter ablation highlighted that as many as 65% might show objective signs of HFpEF, upon invasive testing through right heart catheterization (RHC) and exercise testing [49]. Zakeri et al. observed 939 subjects from 1983 to 2010 and reported that 66% of all HFpEF patients had AF diagnosed either before (29%), during (23%), or after (15%) the diagnosis of HFpEF [50]. These data suggest that a previous history of AF significantly increases the likelihood of new-onset HFpEF [3,51], though this could simply be an epiphenomenon, since AF is easier to diagnose and requires simpler testing than HFpEF [52] and since it may be more symptomatic in patients with concomitant HFpEF, possibly leading to earlier clinical detection [53]. AF in HFpEF is associated with older age, female sex, higher NT-proBNP levels, and larger LA volumes, while new-onset AF after HFpEF is linked to more severe diastolic dysfunction [50]. Despite higher rates of both HF phenotypes among patients with AF, AF independently predicts incident HFpEF—but not HFrEF—after adjustment for shared risk factors [3]. Melenovsky et al. demonstrated that HF phenotypes exhibit distinct patterns of LA maladaptation: HFrEF is characterized by larger atrial volumes and eccentric remodelling, whereas HFpEF shows reduced atrial compliance with higher filling atrial pressures. These abnormalities can precipitate paroxysmal AF and lead to decompensation [54,55].

Overall, AF and HFpEF tend to arise concurrently from a common myocardial substrate, making the apparent order of onset more a reflection of diagnostic timing than causality [27]. With increasing AF burden, LA remodelling, stiffness, and contractility progressively worsen. Consequently, patients with HFpEF and permanent AF exhibit diminished exercise capacity, greater RV dysfunction, and a higher risk of death, compared to HFpEF patients without a history of AF [56].

## 7. Impact of AF on HFpEF Morbidity and Mortality

LA myopathy, and particularly LA stiffness, has been shown to correlate with greater mortality among HFpEF, but not HFrEF patients [54]. Several studies confirmed that AF is significantly associated with reduced survival among HFpEF individuals compared with HFpEF patients in sinus rhythm, even after multivariate adjustment for age, sex, and other relevant clinical variables. This is possibly because AF burden is a reflection of more advanced overall myopathy [3,27,50]. Moreover, a large meta-analysis showed that AF increases the risk of all-cause mortality in HFpEF patients by 11% (HR 1.11, 95% CI 1.09–1.12), while also being an independent predictor of HF-related hospitalization, cardiovascular death, and stroke [57], with similar results reported in an analysis of the TOPCAT trial (Spironolactone for Heart Failure with Preserved Ejection Fraction) [58]. In another post hoc analysis of TOPCAT, Saksena et al. clearly established that any episode of AF (even if paroxysmal) increases the risk of HF progression from NYHA classes I–II to III–IV (HR 1.30, 95% CI 1.04–1.62), and if AF is already present at baseline before HFpEF diagnosis, the relative risk of HF hospitalization is increased significantly by 65% [59]. This increased risk of hospitalization becomes apparent within three months of AF onset and continues to rise over the next five years, accelerating the risk of mortality from heart failure. This trajectory indicates that AF in HFpEF may reflect more advanced disease, rather than act as an independent risk factor [60]. Given the observational nature of the data, this association may be due to confounding factors not accounted for in the matching analysis, suggesting that patients with AF have more comorbidities and that AF may be a marker of higher risk rather than a direct driver of worse prognosis. Because of the high prevalence of AF in HFpEF, systemic embolism and stroke occur more frequently in this group than in patients with AF and HFrEF or HFmrEF (HR, 3.192; 95% confidence interval, 1.039–9.810). No significant differences in the incidence of major bleeding exist across groups [61].

## 8. HFpEF-AF Management

### 8.1. Addressing Comorbidities and Risk Factors

Compared to patients with HFrEF, those with HFpEF are typically older and frailer, which may account for their higher readmission rates for non-cardiac comorbidities (e.g., obesity, COPD, anemia, and hypertension) [62], while cardiovascular-related or HF-specific hospitalizations tend to be higher among HFrEF patients [9]. This emphasizes the need for rigorous comorbidity management in HFpEF to mitigate the deleterious disease mechanisms shared with AF, improve quality of life (QoL), and prevent rehospitalization [5,24,48]. Importantly, addressing comorbidities can also support sinus rhythm maintenance, including in patients with persistent AF [63].

HFpEF patients should ideally have their blood pressure (BP) maintained below 130/80 mmHg [48], although this was not shown to improve outcomes [64]. In overweight or obese patients with HFpEF-AF, a sustained 10% reduction in body weight led to a six-fold lower risk of AF recurrence and improved maintenance of sinus rhythm [65], as shown in the LEGACY trial. Additionally, a reduction in BMI can reduce epicardial fat, lower local myocardial inflammation from adipocytes, reduce remodelling and AF recurrence, and improve haemodynamics and exercise tolerance [66]. Every tenfold increase in exercise has been associated with a 42.8% risk reduction in AF occurrence in HFpEF patients [67]. OSAS should be managed with the help of pneumologists and sleep specialists, as it affects up to 70% of patients with HFpEF and AF [68,69], and CPAP treatment was associated with a lower risk of progression from paroxysmal or persistent AF to permanent AF [70]. Iron deficiency anemia should always be corrected in patients with AF, since it represents an independent risk factor for HF, major bleeding, and death [71], as well as being associated with a higher recurrence of AF after catheter ablation [72]. DM and CKD tend to coexist in patients with HFpEF, with negative synergistic effects on cardiovascular outcomes [73], and CKD-HFpEF patients appear to be at an increased risk of fluid overload and diuretic resistance [74]. Similarly to hypertension, no additional cardiovascular benefits have been demonstrated with intensive versus standard glucose control in people with established type 2 DM [75]. Both DPP4 inhibitors and thiazolidinediones should be avoided in patients with HFpEF because of their potential risk to increase HF hospitalization rates [76,77]. Finally, alcohol reduction in just ≥1% from baseline is associated with a lower risk of AF recurrence 1 year post catheter ablation (HR 0.630 [95% CI, 0.518–0.768]) [78]. However, alcohol has not been shown to have a measurable impact on the HFpEF population [79]. Smoking cessation is of utmost importance in all HFpEF-AF patients.

### 8.2. Medical Treatment of HFpEF

#### 8.2.1. SGLT2 Inhibitors

SGLT2is are continuing to emerge as a promising therapeutic class for improving cardiovascular outcomes in patients with HFpEF. In landmark trials (EMPEROR-Preserved and DELIVER), both empagliflozin and dapagliflozin significantly reduced rates of HF hospitalization and cardiovascular death in patients with HFpEF, regardless of diabetes status (worsening HF incidence dapagliflozin vs. placebo: 11.8% vs. 14.5, respectively, HR 0.79; cardiovascular death: 7.4% vs. 8.3%, respectively, HR 0.88; 95% CI, 0.74–1.05) [80,81]. Importantly, SGLT2is confer pleiotropic and, importantly, renal benefits: they improve patient-reported QoL, as shown in PRESERVED-HF [82], and they exert direct cardioprotective effects at both the ventricular and atrial levels. These effects include reductions in oxidative stress, BP, plasma volume, and epicardial adipose tissue, as well as decreases in PCWP both at rest and during exercise, lower LA pressures, and improved arterial compliance [83,84,85]. Additionally, dapagliflozin might improve systemic arterial compliance and venous capacitance during exercise, providing some evidence for a reduction in arterial wall stiffness, although no statistically significant effect of dapagliflozin was observed on resting BP [86]. Of particular interest in the HFpEF-AF population, SGLT2i may help reduce AF burden through improved LA remodelling, inhibition of the Na^+^/H^+^ exchanger, and reduced atrial AP inducibility [87]. Trials like DECLARE-TIMI 58 and meta-analyses have recently documented a reduction in incident AF with SGLT2i in patients with type 2 diabetes and HF [88,89].

#### 8.2.2. GIP/GLP-1 Receptor Agonists

In the SUMMIT trial, tirzepatide showed a reduction in the risk of cardiovascular death and worsening HF, together with better exercise tolerance in patients with obesity-related HFpEF [90]. This was mainly the result of reduced blood pressure and volume expansion, but also of attenuated systemic inflammation, long-term improvement of eGFR and microalbuminuria, and lower levels of troponin T and NT-proBNP, mirroring a beneficial effect on the myocardium [91]. While a study demonstrated that GLP-1 receptor agonists, as a class, show a neutral overall effect on the risk of developing AF [92], another meta-analysis of 10 RCTs specifically showed that semaglutide (a GLP-1 agonist) can produce a 42% reduction in incident AF compared to placebo (RR 0.58), particularly in individuals with an elevated cardiovascular risk [93].

#### 8.2.3. MRAs

MRAs have shown potential benefit in selected patients with HFpEF, though the existing evidence about their benefits in HFpEF remains controversial. The TOPCAT trial failed to show a significant reduction in the composite endpoint of HF hospitalization or cardiovascular death in HFpEF [94]. However, a regional post hoc analysis suggested improved HF outcomes in selected populations [95]. Importantly, in the TOPCAT trial, 93% of HFpEF patients with AF were on rate control therapy, and the effect of spironolactone did not impact incident AF events, and it was independent of the presence or absence of AF [58]. Spironolactone treatment showed a 21% adjusted relative reduction in mortality, according to an observational study of a sizable American veterans cohort, limiting its applicability to other populations [96]. This benefit did not, however, translate into fewer hospital stays, raising questions about the mechanism by which spironolactone may improve survival. Moreover, in the IMPRESS-AF trial, spironolactone failed to show any improvement in QoL or exercise tolerance in the HFpEF-AF population [97]. Nonetheless, some evidence suggests that MRAs could be more effective in patients belonging to the cardiometabolic HFpEF phenogroup, as they reduce inflammatory cytokine production and improve adipokine profiles [98]. Clearly, the role of MRA is not generalizable across the HFpEF spectrum.

#### 8.2.4. ACE Inhibitors, ARBs, and ARNIs

A meta-analysis of 13 studies by Khan et al. [99] showed that multiple randomized clinical trials of ACE inhibitors and ARBs failed to demonstrate any effect of these drugs on all-cause mortality in patients with HFpEF (RR = 1.02, 95% CI = 0.93–1.11), while observational studies did show a significant improvement in mortality (RR = 0.91, 95% CI = 0.87–0.95, *p* = 0.005). Additionally, multiple trials reported a trend towards lower hospitalization rates when ACE inhibitors and ARBs were administered in the HFpEF population [99]. In the CHARM-Preserved trial, candesartan did not impact mortality endpoints, but it did result in a reduction in HF hospitalizations [100]. Irbesartan, instead, did not affect mortality rates or HF readmissions [101]. Despite early promises and good results in patients with HFrEF, ARNIs have never shown any consistent benefit for HFpEF. A prior phase 2 trial (PARAMOUNT) demonstrated reductions in NT-proBNP and left atrial volume at 12 weeks follow-up, suggesting potential for atrial remodelling [102]. However, in the PARAGON-HF trial, sacubitril/valsartan did not significantly improve AF incidence, HF hospitalizations, and cardiovascular death, although a small trend toward benefit emerged among women and patients with lower EF ranges [103].

#### 8.2.5. Statins

Statins are believed to offer some benefit in HFpEF, owing to their multiple anti-inflammatory and metabolic effects. Observational studies have linked statin use to lower mortality rates in patients with HFpEF, but high-quality evidence is still lacking [104]. Statins may limit endothelial dysfunction by reducing oxidative stress and restoring local nitric oxide availability, with positive effects on diastolic function and ventricular remodelling [105]. Experimental studies further support their role in limiting myocardial fibrosis, reducing epicardial fat, and reversing hypertrophy, all of which are highly relevant in HFpEF pathophysiology [106]. Given their favourable safety profile and positive effects on the endothelial system, statins may be a reasonable adjunct therapy in HFpEF, especially in patients with dyslipidaemia or vascular disease.

#### 8.2.6. Diuretics

Even if diuretics do not improve mortality in HFpEF patients, they can be used to reduce filling pressures and relieve signs and symptoms of congestion, as part of the long-term medical management of these patients with the aim of limiting HF-related hospitalization. However, patients with obesity-related HFpEF may tolerate diuresis more poorly than non-obese HFpEF, with a greater risk of worsening renal function during decongestion, despite the presence of more marked volume expansion [107].

### 8.3. Medical Treatment of AF

#### 8.3.1. Rate vs. Rhythm Control

Considering the negative hemodynamic and substrate-related effects of sustained AF on HFpEF, managing AF with a rhythm control strategy appears to be a reasonable approach to improve cardiovascular outcomes in HFpEF-AF patients [108]. Important results in favour of rhythm control were observed in the EAST-AFNET 4 trial, which showed that early rhythm control of AF (diagnosed within 12 months before enrolment) was associated with a reduction in adverse cardiovascular outcomes compared to rate control [109]. Of note, in the EAST-AFNET 4 trial, about 75% of participants in the rhythm control arm received antiarrhythmic drugs (AADs), while only about 19.4% had undergone catheter ablation (CA) by 2 years of follow-up [110].

The recent SHAM-PVI trial [111] compared CA with placebo (sham procedure, consisting of phrenic nerve pacing) and concluded that CA through pulmonary veins isolation (PVI) led to a statistically significant reduction in AF burden 6 months after the procedure (16.13% absolute reduction in AF episodes in the intervention group), together with a clinically relevant improvement in reported symptoms and QoL. The benefits of ablation relative to AAD have been shown in the EARLY-AF trial [112] and its follow-up study, published in 2023 [113]. It was observed that, over a 3-year follow-up period, cryoballoon ablation as the initial treatment of paroxysmal AF resulted in a lower incidence of persistent or recurrent AF compared to AADs. At the end of the study, only 5.2% of patients in the cryoablation group had been hospitalized, compared with 16.8% in the AAD group (RR = 0.31; 95% CI: 0.14–0.66). Moreover, a lower incidence of persistent AF was present in the CA group compared to the AAD group (1.9% vs. 7.4%, respectively) [113].

#### 8.3.2. Catheter Ablation in HFpEF

Unlike patients with HFrEF, for whom numerous randomized trials have validated the role of catheter ablation, randomized evidence for HFpEF is still missing. The CASTLE-AF and CASTLE-HTx trials demonstrated that CA is superior to medical therapy for the management of AF in patients with HFrEF, resulting in a greater improvement in LVEF, QoL, and survival [114,115]. A large randomized trial investigating the effects of CA for AF in patients with HFpEF and HF with mildly reduced EF on cardiovascular death and hospitalizations is, instead, currently ongoing (CABA-HFPEF), and another one will investigate the role of the “ablate and pace” strategy in patients with HFpEF or HFmrEF and permanent AF (PACE-FIB [116]). However, while awaiting randomized evidence—which remains the only way to definitively establish the role of catheter ablation in this subset of patients—several observational studies and sub-analyses of randomized controlled trials have already explored the role of catheter ablation in the HFpEF population.

HFpEF is a known risk factor for AF recurrence after catheter ablation in the general population, since LA myopathy may be consequent to other etiologies; nevertheless, catheter ablation still shows beneficial effects compared to medical therapy for the control of the arrhythmia in this cohort of patients [117,118]. In a prespecified CABANA sub-analysis involving 778 patients with AF and stable HF (79% HFpEF), CA proved superior to AAD, yielding better survival, less AF recurrence, and improved quality of life. CA was associated with a reduction in the primary composite outcome by 36% and all-cause mortality by 43% [119]. In addition, both the MANTRA and ATTEST trials further confirmed the superiority of CA over AAD in limiting AF burden and recurrence [120,121].

To the best of our knowledge, only one small randomized controlled trial exists that is specifically dedicated to the HFpEF-AF population (Table 2). This study demonstrated that AF ablation may significantly improve exercise hemodynamic parameters and QoL. Remarkably, at 6-month follow-up, up to 50% of patients who underwent ablation no longer met the hemodynamic diagnostic criteria for HFpEF, suggesting that successful rhythm control may reverse functional and structural abnormalities common to HFpEF and AF. Instead, only <10% of HFpEF patients in the medical therapy arm experienced similar benefits [122]. Complementing these findings, a large propensity-matched retrospective study reported that CA was associated with a 56% relative reduction in mortality and a significant decrease in acute HF hospitalizations compared to AAD [123]. Similarly, a systematic review and meta-analysis by Bulhões et al. involving 20.000 patients further confirmed that CA was linked with significantly lower risks of all-cause mortality, cardiovascular death, HF hospitalization, and all-cause readmission when compared with medical therapy (either rate or rhythm control), but neutral effects were seen on stroke and TIA rates [124]. Other single-centre and prospective studies further support these observations, showing improvements in HFpEF-AF patients in terms of diastolic function, NT-proBNP levels, NYHA functional class, and echocardiographic parameters post-CA, with up to one-third of patients no longer fulfilling the criteria for HFpEF at follow-up [49,119,125,126,127]. In another investigation, CA also seemed to exert a positive impact on the atria, with significantly improved LA diameter and NT-proBNP levels in the post-ablation period, as well as on the incidence of stroke or systemic embolism, which was significantly reduced in patients undergoing ablation [128]. These associations have also been observed in pulsed field ablation, as the MANIFEST-PF registry further supports the potential role of PFA in HFpEF-AF. Based on data from this large registry, PFA was found to be safe and effective across HF phenotypes, with no increase in procedural complications and with favourable arrhythmia-free survival at one year [129]. These findings are limited by the observational nature of the data; indeed, selection bias may be present, and the positive results may be limited to a subset of patients in whom LA myopathy represents the primum movens of HFpEF rather than merely a consequence of the disease.

In fact, in stark contrast with these data, other observational studies reported different findings (Table 2). A meta-analysis including data derived from sub-analyses of 3 RCTs and a total of 913 patients with HFpEF showed that even though CA was associated with reduced risk of HF readmission among patients with HFrEF, this association was not present in AF patients with HFpEF (risk ratio—RR: 0.93, 95% CI: 0.65–1.32); in addition, cardiovascular death was not significantly reduced (RR: 0.91; 95% CI: 0.46–1.79) [133]. Another study, comparing patients with HFpEF-AF versus AF-only patients without any history of HF, found that patients with HFpEF experienced AF recurrence more often compared to AF-only patients (57% versus 23%; *p* = 0.003) and were more susceptible to AF-related rehospitalization (26% versus 7%; *p* = 0.016). Additionally, HF symptoms and elevated NP levels persisted even among those HFpEF patients still in rhythm control at follow-up, with no change in QoL [134]. These findings may stem from the heterogeneity in HFpEF definitions, the low proportion of HF hospitalizations among overall events, and the higher comorbidity burden characteristic of HFpEF. As noted above, a new episode of AF is an independent risk factor for worsening HF (HR 1.30) and for mortality due to heart failure in more advanced HFpEF [59]. These data indicate that the prognosis of HFpEF should be reassessed whenever incident AF occurs, and that restoration of sinus rhythm in subjects with HFpEF could lower the relative risk of future HF by 60% (from 25% to 10%). Equally, achieving a long-term reduction in AF burden through rhythm control may interrupt the HFpEF-AF vicious cycle, potentially modifying the natural course in individuals with prevalent disease [60]. These observations are hypothesis-generating and should be confirmed by randomized evidence.

Moreover, rate control strategies should be used with caution in patients with HFpEF, in contrast with HFrEF patients, in whom beta-blockers represent one of the pillars of medical therapy, as they may exacerbate chronotropic incompetence and, through excessive prolongation of diastole, contribute to increased filling pressures [135,136]. In line with this, withdrawal of beta-blocker therapy in stable HFpEF patients with chronotropic incompetence has been shown to significantly improve maximal functional capacity, as measured by peak oxygen consumption (peak VO_2_) [136]. Regardless of which technique is chosen, anticoagulation should be administered to HFpEF-AF patients without any absolute contraindication to anticoagulation according to the CHA2DS2-VA score. It should be noted that HFpEF, like HFrEF, should be considered as “Congestive heart failure” within the CHAD2DS2-VA score; moreover, since patients with HFpEF present more comorbidities, indication to anticoagulation is frequent in this cohort [137].

#### 8.3.3. Selecting Patients for Ablation

CA in HFpEF-AF patients requires careful consideration, as these patients typically have more comorbidities and are at higher risk of procedural failure. As such, timely intervention in patients with paroxysmal or new-onset AF, through aggressive risk factor modification, treatment of comorbidities, and early rhythm control (CA) to preserve LA compliance and prevent progression to advanced LA and LV myopathy, is crucial. Therefore, to guide patient selection, thorough pre-procedural risk stratification, including LA strain assessment, AF burden, and evaluation of comorbidity, is recommended.

Finally, because of the older age, increased frailty, and high prevalence of comorbidities typical of the HFpEF population, the procedural risk-benefit ratio must always be critically assessed prior to CA, especially in patients with limited functional capacity.

### 8.4. New Experimental Strategies

Several experimental and device-based strategies are currently under study to address the complex interplay between AF and HFpEF.

One such approach is interatrial shunting, aimed at reducing elevated LA filling pressure and decreasing pulmonary congestion. The REDUCE LAP-HF II trial evaluated the impact of an atrial shunt device in patients with HFpEF, but it failed to demonstrate any significant benefits in terms of cardiovascular death, stroke, HF readmissions, or improved QoL [138]. An important finding emerging from the REDUCE LAP-HF trial is that patients with elevated exercise pulmonary vascular resistance (PVR), defined as invasively measured PVR > 1.74 WU, experienced worsening right heart haemodynamics, whereas those with normal pulmonary vascular response to exercise showed potential benefit. Wireless pulmonary artery pressure monitoring, using implantable sensors such as CardioMEMS, represents another promising approach. In the CHAMPION trial, CardioMEMS led to a 50% reduction in HF hospitalizations in patients with HFpEF, highlighting the importance of hemodynamic-guided management in this population [139]. Lastly, research into targeted molecular therapies that aim to disrupt the fibrotic and inflammatory pathways that drive the pathogenesis of both HFpEF and AF continues, demonstrating that pharmacologic innovation is still a frontier (see Table 5 in [5], Borlaug et al.).

## 9. Conclusions

The HFpEF-AF overlap syndrome is highly prevalent and poses substantial diagnostic and therapeutic challenges, and high-quality evidence to guide optimal management remains limited. Additional randomized trials are needed to define the role of catheter ablation and to identify the patients most likely to benefit. Early AF detection, risk stratification, and targeted management of modifiable risk factors are essential to improving outcomes and slowing disease progression.

## Figures and Tables

**Table 1:** Clinical HFpEF phenogroups as identified by Larson et al. [14]. It is important to note that such phenogroups show extensive overlap, and individuals belonging to >1 class show an increased risk of death and HF-related hospitalization.

HFpEF Phenogroup and Estimated Prevalence	Main Features
Cardiometabolic (72%)	Patients with diabetes and/or with a BMI > 30 kg/m^2^
Stiff vascular (57%)	Patients with a pulse pressure at rest > 90 mmHg or TACI < 0.5 mL/m^2^
LA myopathy (53%)	Patients with LAVI > 34 mL/m^2^ or with AF
Pulmonary vascular disease (40%)	Patients with resting mPAP > 20 mmHg and resting PVR > 2 WU
No phenogroup (5.7%)	

Abbreviations: HFpEF = Heart Failure With Preserved Ejection Fraction; AF = Atrial Fibrillation; LA = Left Atrium; BMI = Body Mass Index (kg/m^2^); TACI = Total Arterial Compliance Index; LAVI = Left Atrial Volume Index; mPAP = mean Pulmonary Artery Pressure; PVR = Pulmonary Vascular Resistance; WU = Wood Unit.

**Table 2:** Overview of studies investigating the effects of CA on AF in HFpEF-AF patients.

Paper	Level of Evidence	Sample Size	Outcomes (QoL, Imaging, HFpEF Defining Criteria)	Impact on HHF and Mortality
**2a.** Overview of studies investigating the effects of CA on AF in HFpEF-AF patients (positive results).				
Chieng et al. (2023) [122]	RCT	31 total (16 ablation, 15 medical therapy)	Improved peak exercise PCWP (~30.4 → ~25.4 mm Hg). Increase in peak VO_2_ (≈20.2 → 23.1 mL/kg/min). Lower NT-proBNP levels (from ~794 → ~141 ng/L). Improved QoL: MLHF score improved significantly. 50% of the ablation group no longer fulfilling criteria for HFpEF (based on invasive RHC) vs. 7% medical therapy arm at 6 months.	Not clearly powered for long-term readmission/mortality; 6-month follow-up focused on hemodynamics, symptoms, and biomarkers.
Xie et al. (2023) [130]	Observational(propensity matched)	1034 HFpEF-AF patients; 392 first-time ablation, 642 medical therapy	Reduced AF/AT recurrence (~33% lower risk in ablation group).QoL/symptoms: implied via reduced hospitalizations and maintaining sinus rhythm.	Composite endpoint (HF hospitalization, death) significantly lower in the CA group: aHR 0.55 (95% CI 0.37–0.82), *p* = 0.003, mostly hospitalizations.
Liu et al. (2024) [131]	Retrospective observational	116 HFpEF patients	Significant decrease in LA size at 1 year post-ablation Increased LVEF in the HFpEF group. Improvements in LA strain and LA storage and pump function; NO change in left atrial conduit function. (E/e′) did NOT improve significantly (E/e′ baseline ≈14.11 vs. 14.30 at 1 year; *p* = 0.85).	N/A
Martens et al. (2025) [117]	Post hoc analysis of CABANA (RCT)	CABANA: 1763 patients (H_2_FPEF score: ≥6)	Greater treatment effect of ablation in reducing AF recurrence in the high HFpEF probability group vs. the lower score group; interaction *p* = 0.035. Improvement in NYHA class greater in those with a high probability of HFpEF after AF ablation.	Composite endpoint (HF hospitalization, death) significantly lower in the CA group: (HR 0.82; *p* = 0.025), mostly hospitalizations.
Yamauchi et al. (2021) [126]	Prospective observational	1173 patients with NON-paroxysmal AF; 293 HFpEF patients	AF recurrence at 1 year: in HFpEF, 48/293 (≈16.4%) recurrence; sinus rhythm maintained in ~94.8% at 1 year. Significant improvement in LA diameter in the HFpEF group. NYHA functional class improved. BNP levels significantly decreased from baseline to 1 year.	N/A
Rattka et al. (2021) [125]	Observational propensity matched	127 (43 ablation vs. 43 AAD after matching)	Improved echocardiographic parameters and improved diastolic function; 35% of the CA group no longer met HFpEF diagnostic criteria vs. 9% in the medical therapy group. Reduced AF recurrence.	Composite endpoint (HF hospitalization, death) significantly lower in the CA group: HR 0.30 (95% CI 0.13–0.67), mostly hospitalizations.
**2b.** Overview of studies investigating the effects of CA on AF in HFpEF-AF patients (negative/mixed results).				
Nagai et al. (2019) [127]	Observational before-and-after	30 patients (persistent AF, no recurrence after RFCA)	LAVI decreased (33.7 ± 10.4 → 24.6 ± 8.6 mL/m^2^) at 6 months. LVEF improved (56.8% ± 9.8 → 65.1% ± 9.1). Diastolic indices: strain rate during early diastole (SR_e_) improved (0.73 ± 0.10 → 1.32 ± 0.29 s^−1^). Ratio E/SR_e_ decreased (1.11 ± 0.36 → 0.61 ± 0.19), indicating improved LV filling pressure/diastolic function.	N/A
Long et al. (2025) [128]	Retrospective Observational	570 patients: 187 HFpEF + CA, 187 HFpEF + AAD, 196 without HFpEF + ablation	SR maintenance: 50.8% with CA vs. 12.5% with AAD (*p* < 0.001). LVEDD, NYHA class, LVMI, LAVI improved with CA vs. AAD (*p* < 0.001). Stroke incidence: 9.63% with CA vs. 16.58% with AAD (*p* < 0.01).	Mortality: 7.5% with CA vs. 12.8% with AAD (*p* = 0.49). HF hospitalizations: 0.38 per patient in the CA arm vs. 1.28 in the AAD arm (*p* < 0.001).
Machino-Ohtsuka et al. (2013) [132]	Prospective observational	40 patients	SR maintenance: 80% at 12 months. Significant reduction in LAVI (from 51.2 ± 12.3 to 45.8 ± 11.2 mL/m^2^, *p* < 0.01). Improvement in LVEF (from 58.2 ± 7.3% to 61.5 ± 7.5%, *p* < 0.05). Decrease in NT-proBNP levels (from 1200 ± 800 to 800 ± 600 pg/mL, *p* < 0.01).	N/A
Oraii et al. (2023) [133]	Systematic review and meta-analysis	3 RCTs with 2465 participants (1552 HFrEF, 913 HFpEF)	No significant difference in HF events between ablation and conventional therapies in HFpEF (RR 0.93; 95% CI 0.65–1.32).	No significant difference in cardiovascular death between ablation and conventional therapies in HFpEF (RR 0.91; 95% CI 0.46–1.79). No significant difference in all-cause mortality between ablation and conventional therapies in HFpEF (RR 0.95; 95% CI 0.39–2.30).
Zylla et al. (2022) [134]	Prospective observational	102 AF patients (24 with HFpEF)	HF symptoms and elevated NT-proBNP persisted, even in patients with successful rhythm control at follow-up. Echocardiographic follow-up showed progression of adverse left atrial remodelling and no relevant improvement in diastolic function in HFpEF. QoL improved in patients without HFpEF, whereas patients with HFpEF still exhibited a lower physical component summary score (median, 41.5 versus 53.4; *p* < 0.004).	N/A
Nagai et al. (2019) [127]	Observational before-and-after	30 patients (persistent AF, no recurrence after RFCA)	LAVI decreased (33.7 ± 10.4 → 24.6 ± 8.6 mL/m^2^) at 6 months. LVEF improved (56.8% ± 9.8 → 65.1% ± 9.1). Diastolic indices: strain rate during early diastole (SR_e_) improved (0.73 ± 0.10 → 1.32 ± 0.29 s^−1^). Ratio E/SR_e_ decreased (1.11 ± 0.36 → 0.61 ± 0.19), indicating improved LV filling pressure/diastolic function.	N/A

Abbreviations: AAD: anti arrhythmic drugs; AF: atrial fibrillation; AT: atrial tachycardia; BNP: B-type natriuretic peptide; CA: catheter ablation; HF: heart failure; HHF: hospitalizations for heart failure; LA: left atrium; LAVI left atrium volume indexed; LVEDD: left ventricular end-diastolic diameter; LVEF: left ventricular ejection fraction; LVMI: left ventricular mass index; MLHF: Minnesota Living with Heart Failure; N/A: not available; PCWP: pulmonary capillary wedge pressure; QoL: quality of life; RCT: randomized controlled trial; RFCA: radiofrequency catheter ablation; RHC: right heart catheterization; SR: sinus rhythm.

## Data Availability

No new date were created or analyzed in this study. Data sharing is not applicable to this article.
